# Oxygen therapy for acute myocardial infarction

**DOI:** 10.1590/S1516-31802010000600013

**Published:** 2010-12-02

## Abstract

**BACKGROUND:**

Oxygen (O2) is widely recommended for patients with myocardial infarction yet a narrative review has suggested it may do more harm than good. Systematic reviews have concluded that there was insufficient evidence to know whether oxygen reduced, increased or had no effect on the heart ischaemia or infarct size.

**OBJECTIVE:**

To review the evidence from randomized controlled trials to establish whether routine use of inhaled oxygen in acute myocardial infarction (AMI) improves patient-centered outcomes, in particular pain and death.

**CRITERIA FOR CONSIDERING STUDIES FOR THIS REVIEW:**

The following bibliographic databases were searched (to the end of February 2010): Cochrane Central Register of Controlled Trials (CENTRAL) (The Cochrane Library), Medline, Medline In-Process, Embase, CINAHL, Lilacs and PASCAL, British Library ZETOC, Web of Science ISI Proceedings. Experts were also contacted to identify any studies. No language restrictions were applied.

**SELECTION CRITERIA:**

Randomized controlled trials of people with suspected or proven AMI, less than 24 hours after onset, in which the intervention was inhaled oxygen (at normal pressure) compared to air and regardless of co-therapies provided these were the same in both arms of the trial.

**DATA COLLECTION AND ANALYSIS:**

Two review authors independently reviewed the titles and abstracts of identified studies to see if they met the inclusion criteria and independently undertook the data extraction. The quality of studies and the risk of bias were assessed according to guidance in the Cochrane Handbook. The primary outcomes were death, pain and complications. The measure of effect used was the relative risk (RR).

**MAIN RESULTS:**

Three trials involving 387 patients were included and 14 deaths occurred. The pooled RR of death was 2.88 (95% CI 0.88 to 9.39) in an intention-to-treat analysis and 3.03 (95% CI 0.93 to 9.83) in patients with confirmed AMI. While suggestive of harm, the small number of deaths recorded meant that this could be a chance occurrence. Pain was measured by analgesic use. The pooled RR for the use of analgesics was 0.97 (95% CI 0.78 to 1.20).

**AUTHORS’ CONCLUSIONS:**

There is no conclusive evidence from randomized controlled trials to support the routine use of inhaled oxygen in patients with acute AMI. A definitive randomized controlled trial is urgently required given the mismatch between trial evidence suggestive of possible harm from routine oxygen use and recommendations for its use in clinical practice guidelines.

**FURTHER INFORMATION:** Centro Cochrane do Brasil Rua Pedro de Toledo, 598 Vila Clementino — São Paulo (SP) — Brasil CEP 04039-001 Tel. (+55 11) 5579-0469/5575-2970 http://www.centrocochranedobrasil.org.br/

**This section was edited under the responsibility of the Brazilian Cochrane Center.**

**For Latin America and the Caribbean, the full text of the review is freely available from:** http://cochrane.bvsalud.org/cochrane/main.php?lang=pt&lib=COC

**For other regions, the abstract is available (through the Cochrane Journal Club) from:** http://www.cochranejournalclub.com/oxygen-therapy-for-acute-myocardial-infarction-clinical/

## COMMENTS

Some medical management methods today are still based on habits, customs and experiences. However, in the era of evidence-based medicine, it is not enough for a medical approach to seem logical and have a physiopathological basis. Scientific development demands that its efficacy and effectiveness should be proven. In general, this is only possible through intervention studies (clinical trials) and, when there are divergences between these studies, it is recommended that they should be combined through a meta-analysis produced from a systematic review.

In this manner, in a systematic review on the use of oxygen therapy in cases of acute myocardial infarction (AMI), Cabello et al.^1^ reveal a scenario in which a classical management method that until now has been used without questioning is shown to lack a scientific basis and present potential risk. After an exhaustive search in the medical literature, they identified only three studies with a total sample of 387 patients. Because of methodological problems relating to selection criteria, identification of primary and secondary outcomes (death, chest pain and size of AMI size), dropouts and randomization, they conclude that there is a high chance of bias. Consequently, the meta-analysis on the data does not allow any categorical conclusion, except that there is no evidence that validates this approach, which is still widely used in coronary units. Hence, there is an urgent need to conduct studies that might clarify the usefulness of oxygen therapy for AMI cases. The immediate impact of this review is that if there is no clinical indication for using oxygen, it should not be prescribed.
